# Deep learning for screening primary osteopenia and osteoporosis using spine radiographs and patient clinical covariates in a Chinese population

**DOI:** 10.3389/fendo.2022.971877

**Published:** 2022-09-13

**Authors:** Liting Mao, Ziqiang Xia, Liang Pan, Jun Chen, Xian Liu, Zhiqiang Li, Zhaoxian Yan, Gengbin Lin, Huisen Wen, Bo Liu

**Affiliations:** ^1^ Department of Radiology, The Second Affiliated Hospital of Guangzhou University of Chinese Medicine, Guangzhou, China; ^2^ Department of AI Research Lab, Guangzhou YLZ Ruitu Information Technology Co, Ltd, Guangzhou, China; ^3^ Department of Radiology, ZHUHAI Branch of Guangdong Hospital of Chinese Medicine, Zhuhai, China

**Keywords:** osteoporosis, convolutional neural network (CNN), screening, dual-energy x-ray absorptiometry (DXA), lumbar spine x-rays

## Abstract

**Purpose:**

Many high-risk osteopenia and osteoporosis patients remain undiagnosed. We proposed to construct a convolutional neural network model for screening primary osteopenia and osteoporosis based on the lumbar radiographs, and to compare the diagnostic performance of the CNN model adding the clinical covariates with the image model alone.

**Methods:**

A total of 6,908 participants were collected for analysis, including postmenopausal women and men aged 50–95 years, who performed conventional lumbar x-ray examinations and dual-energy x-ray absorptiometry (DXA) examinations within 3 months. All participants were divided into a training set, a validation set, test set 1, and test set 2 at a ratio of 8:1:1:1. The bone mineral density (BMD) values derived from DXA were applied as the reference standard. A three-class CNN model was developed to classify the patients into normal BMD, osteopenia, and osteoporosis. Moreover, we developed the models integrating the images with clinical covariates (age, gender, and BMI), and explored whether adding clinical data improves diagnostic performance over the image mode alone. The receiver operating characteristic curve analysis was performed for assessing the model performance.

**Results:**

As for classifying osteoporosis, the model based on the anteroposterior+lateral channel performed best, with the area under the curve (AUC) range from 0.909 to 0.937 in three test cohorts. The models with images alone achieved moderate sensitivity in classifying osteopenia, in which the highest AUC achieved 0.785. The performance of models integrating images with clinical data shows a slight improvement over models with anteroposterior or lateral images input alone for diagnosing osteoporosis, in which the AUC increased about 2%–4%. Regarding categorizing osteopenia and the normal BMD, the proposed models integrating images with clinical data also outperformed the models with images solely.

**Conclusion:**

The deep learning-based approach could screen osteoporosis and osteopenia based on lumbar radiographs.

## Introduction

Osteoporosis is a popular metabolic skeletal disorder with characteristics of low bone mineral density (BMD) and thinning of bone trabecula, leading to enhancement of bone fragility and increased risk of fracture (1). Primary osteoporosis is quite common in the elderly. According to a recent nationwide and multicenter investigation in China, among people over 50 years, the rates of osteoporosis were 29.13% and 6.46% for women and men, respectively (2), which are estimated to increase to 39.2% and 7.5%, respectively, by 2050 (3). At present, it has been estimated that a total of 10.9 million men and 49.3 million women suffer from osteoporosis in China (3). Osteopenia, as a precursor of osteoporosis, is also an important risk factor for fragility fractures. Previous studies have indicated that most women who suffer from fragility fractures have been diagnosed with osteopenia (4, 5). However, the majority of osteoporosis and osteopenia cases are undiagnosed until they experience a fracture, which would lead to a high probability of complications and mortality (6, 7). Hence, early detection of osteoporosis and osteopenia is significant to disease prevention and control, which may prevent osteoporotic fractures and lower the burden of this disease.

BMD value is a credible means for the early detection of osteoporosis and osteopenia. Currently, DXA is recognized as the gold standard for diagnosing osteoporosis and osteopenia globally (8). However, due to inaccessibility, knowledge deficits for screening, and high-cost factors of DXA, the application of DXA is limited. As a result, only a few developing countries are using DXA (9). In China, only 2.8% of people aged ≥20 years have undergone testing, while the rate is 3.7% among those aged ≥50 years (10). DXA-based measures of BMD are the sum of cortical bone and cancellous bone, considering two-dimensional structures, which cannot fully explain the geometry, size, and microstructure of bone (11, 12). It is necessary to explore effective, safe, and cost-balanced substitutes to improve the above situations. Routine lumbar spine x-ray examinations are widely attainable at most hospitals globally. The lumbar spine (LS) radiographs that are ordered for other indications potentially contain useful information about BMD. Utilizing these LS x-ray images to assess BMD synchronously requires no added scanning time, radiation, or additional cost. Thus, this method would be more acceptable to people. However, there were many challenges to evaluating BMD by LS x-ray images, and only a few multicenter studies have been reported presently, which just takes into consideration postmenopausal women aged ≥50 years (13).

In recent years, the deep learning technique represented by the convolutional neural network (CNN) has achieved great success in radiological imaging diagnosis (14, 15). It has been reported that the deep learning technique has been successfully applied to the evaluation of radiological images, such as the differential diagnosis of diseases (16, 17), skeletal maturity assessed by pediatric hand radiographs (18), and the detection of fractures (19–21). This technique has also been applied to aid osteoporosis diagnosis. Numerous modalities have been used: dental radiographs (22), spine radiographs (13, 23), hand and wrist radiographs (24), DXA imaging (25), and spine CT (26, 27). Though some reports are available on osteoporosis diagnosis from spine radiographs using CNN (13, 23), these studies not only have a small number of cases but also did not consider men and clinical covariates. We hypothesized that combining clinical risk factors with image features would improve the models’ capability for diagnosing osteoporosis and osteopenia.

The purpose of this study was to screen osteoporosis and osteopenia with LS x-ray images using CNN in postmenopausal women and men ≥50 years, and to explore whether adding clinical covariates improves the diagnostic performance over the image model alone.

## Materials and methods

### Patient cohort

This retrospective, multicenter study was conducted in a hospital with four independent sub-districts and another large tertiary center in China. The study had been approved by the institutional review board and the ethics committee of the host hospital (Ethics Committee of Guangdong Provincial Hospital of Chinese Medicine ZE2020-299-01), and the informed consent was waived. All images were de-identified before using to protect the privacy of the patients. The clinical and image data of all participants were retrospectively collected from July 2011 to March 2021. Inclusion criteria were as follows: (1) postmenopausal women over 50 years (the menopausal age was identified by medical records or patients’ statement) and men aged over 50 years; (2) all patients had performed both LS x-rays and DXA examinations within 3 months, and had not accepted therapies influenced by BMD; and (3) plain radiographs of LS including anteroposterior (AP) and lateral (LAT) images that must clearly show the first to the fourth lumbar vertebrae. Exclusion criteria were as follows: (1) patients with postoperative metal or bone cement implant of the LS (L1–L4); (2) patients who experienced secondary osteoporosis (such as osteoporosis in renal failure, diabetes, and hyperparathyroidism) or lesions, including tumors and inflammatory diseases; (3) patients with serious scoliosis or deformity; (4) patients with vertebral compression fracture (any vertebrae of L1–L4); and (5) images that show low signal-to-noise ratio affecting to outline the region of lumbar vertebrae.

**Figure 1:**
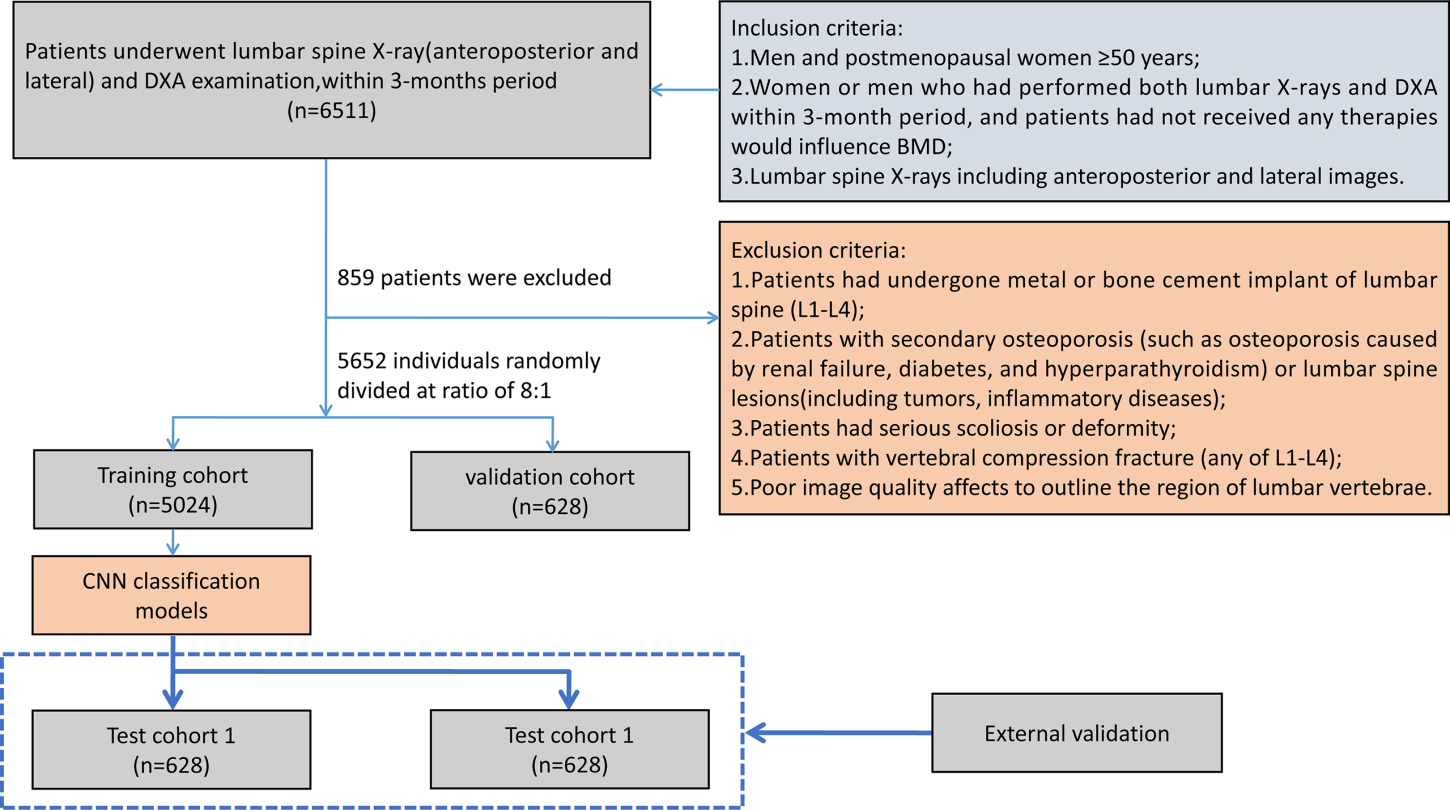
Flowchart of patient selection. BMD, bone mineral density; DXA, dual-energy x-ray absorptiometry; CNN, convolutional neural network.

In total, 6,908 patients who satisfied all criteria were included in the study. A total of 5,652 patients from the three sub-districts between July 2011 and September 2020 were randomly divided into a training cohort and a validation cohort at a ratio of 8:1, and another 628 patients obtained from another independent sub-district between July 2011 and September 2020 were used as test cohort 1; for test cohort 2, 628 patients from another participating center were collected between March 2019 and March 2021. All cases used the same inclusion and exclusion criteria. **Figure 1** shows the flowchart of case selection in different participating centers.

### Study design

The purpose of this study is to develop artificial intelligence models to classify primary osteoporosis and osteopenia from LS radiographs, and the T-scores of LS obtained from DXA examination were used as a reference standard. According to the WHO criteria, all subjects were classified into three categories: osteoporosis defined as T-score ≤ −2.5; osteopenia: −1 > T-score > −2.5; and normal: T-score ≥ −1 (28). T-scores were computed referring to the BMD dataset of young Chinese female or male patients aged 20–40 years. We attempted to develop artificial intelligence models based on CNN through a single channel (AP or LAT images were input respectively) and two channels (AP and LAT images are input simultaneously). Furthermore, we add the clinical data (including sex, age, and BMI) to explore whether it can improve the diagnostic performance of the model.

### Lumbar vertebra radiographs and BMD measurement

In the training and validation cohorts, the lumbar x-ray examinations were performed by the AXIOM Aristos MX/VX Digital Radiographic (DR) apparatus (Siemens, Germany), with parameters set at 70 kVp for AP imaging and 77 kVp for LAT imaging. In test cohort 1, the images were conducted by the Yiso DR apparatus (Siemens, Germany), and 75 kVp was set for AP and 80 kVp for LAT imaging. In test cohort 2, the lumbar x-ray scans were operated by Revolution XR/d DR apparatus (General Electrical, America), with settings at 75 kVp for AP imaging and 90 kVp for LAT imaging. The mAs were automatically adjusted according to body size for all images.

For all participants, the BMD values of lumbar spine were measured using the dual-energy x-ray absorptiometry (Discovery A, HOLOGIC, USA). The patients’ weight and height were measured by the electronic weigher, and the BMI was calculated. The age, weight, and height of patients were acquired from DXA examination records.

### Image preprocessing

The pre-processing of images included three steps. Firstly, all the regions of interest (ROIs) were delineated on lumbar vertebrae (L1–L4) from AP and LAT images, and the specific method was as follows: we used the smallest rectangular frame to include the vertebral body, with lateral margin within 2 mm of the edge of the vertebral body, while the upper and lower edges are in the middle of the intervertebral space. All images were delineated by six radiologists with 4–8 years of experience. Secondly, all ROIs were cropped and then each ROI was resized to 512 × 512 pixels. The filling scale that using gray filling for the blank area is adopted to avoid the lumbar vertebrae being deformed and features destroyed. Finally, in consideration of the differences in x-ray scanning parameters, grayscale normalization was performed in all images to enhance their robustness; Gaussian filtering, histogram equalization, and pixel value normalization were also performed.

### Development of the CNN models in the training cohort

**Figure 2:**
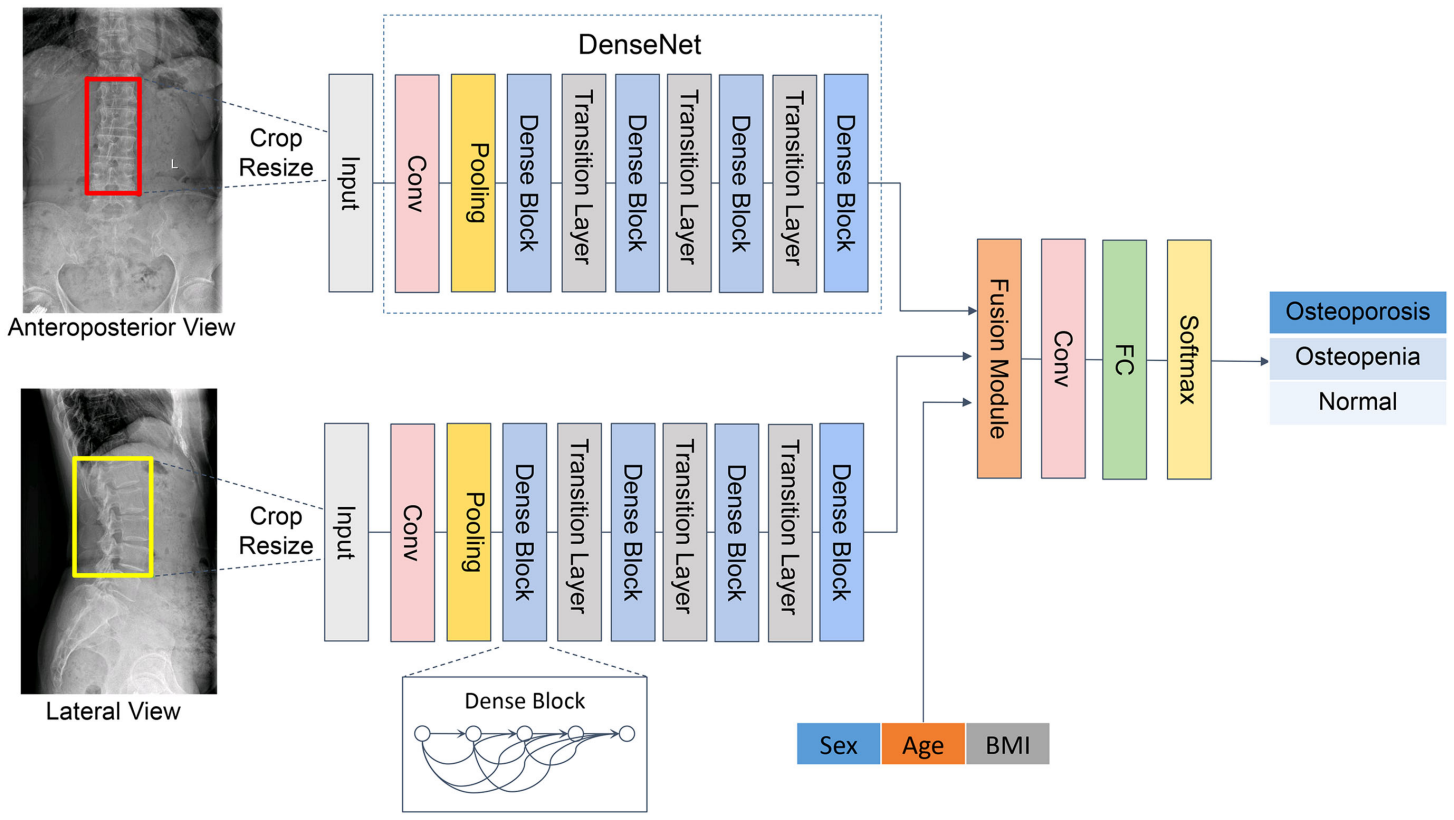
Overview of our proposed framework.

The Dense Convolutional Network (DenseNet) (29) was applied in the backbone network, comprising four dense blocks and three transition layers (**Figure 2**). Each dense block consists of three consecutive operations: batch normalization, followed by a rectified linear unit (ReLU) and a 3 × 3 convolution (Conv). To reduce the number of input feature maps, a 1×1 convolution was introduced as a bottleneck layer before each 3×3 convolution to improve computational efficiency. The layers between blocks were called transition layers, which were used for convolution and pooling. To further enhance the compactness of the model, we reduced the amount of feature maps at transition layers. Following the last dense block, a full connection (FC) is implemented and then a softmax classifier is attached.

The developed CNN classification model is composed of two channels to carry out auto-analysis of the AP and LAT lumbar vertebra (L1–L4) images. Both channels presented the same structure as mentioned above. The features were extracted through DenseNet, which connected each layer to every other layer in a feed-forward pattern. Through skip connection, each layer in the network was directly connected to the previous layer, which strengthened the transmission of features and thus realized the integration of information flow. For each layer, the feature maps of all preceding layers served as a single input, and the features generated from the current layer were input to the subsequent layers. Thus, it could control the vanishing-gradient problem, enhance feature propagation, emphasize feature reuse, and considerably decrease the quantity of parameters.

Since this was a three-category mission, we developed a three-classification CNN model to perform classification from AP, LAT, and AP+LAT views. The results of each case were output from a single channel and from two channels.

### Evaluating the performance of the classification models

A total of 5,652 participants were randomly allocated to training data and validation data at a ratio of 8:1. Independent patients (628 patients) from another sub-district of the same hospital and the other participating center (628 patients) were used as test cohorts that were not included in the training cohorts. The training cohort was used for model development, the validation cohort was employed to filter hyper-parameters and select the best model, and the test cohorts were applied to evaluate the predictive performance of the trained models. The constructed model ultimately classified the patients into osteoporosis, osteopenia, and normal bone mass. Besides image features, we also added clinical covariates (gender, age, and BMI) to the CNN model to explore whether these covariates could improve the performance of the model.

### Statistical analysis

Descriptive statistics were expressed as numbers, and continuous variables were expressed as means ± standard deviations (SDs). Categorical variables were compared by using the chi-square test. *p* < 0.05 was considered a statistically significant difference. The receiver operating characteristic (ROC) curve was used to access the diagnostic effectiveness of the CNN models; meanwhile, the area under the curve (AUC) values and 95% confidence intervals (CIs) for sensitivity and specificity were calculated. We used DeLong’s method for assessing the statistical difference of AUC between different models. In addition, the positive predictive value (PPV) and negative predictive value (NPV) were counted. Moreover, the amount of true positives, false positives, false negatives, and true negatives were demonstrated with the confusion matrix.

All the deep convolutional models were complemented by PYTHON (3.6.6, Guido van Rossum, Netherlands). All statistical analyses were carried out by R software (3.0.2, R Core Team, 2013) and MedCalc software (15.6.1, Microsoft Partner, 2015). All experiments were performed under Windows on a machine with an Intel (R) Core (TM) Processor i7-8700 @ 3.20 GHz central processing unit (CPU), an NVIDIA GeForce GTX graphics processing unit (GPU), and a RAM of 64 GB.

## Results

### Patient demographics

A total of 6,908 patients [mean age, 65.4 years ± 9.3 (SD); range, 50–95 years] including 13,816 lumbar vertebra x-ray images were available for the final analysis. **Table 1** lists the clinical and demographic parameters for the training, validation, and two test cohorts. Gender, age, and BMI among the training, validation, and test cohorts demonstrated no statistically significant differences.

**Table 1:** Demographic characteristics of 6,908 participants.

Characteristics	Training cohort	Validation cohort	Test cohort 1	Test cohort 2	Total
Patients (*n*)	5024	628	628	628	6,908
Age, years, mean (SD)	65.3 (9.2)	65.6 (9.4)	65.3 (9.3)	65.6 (10.0)	65.4 (9.3)
Sex					
Male	1,594	196	190	169	2,149
Female	3,430	432	438	459	4,759
BMI, kg/m^2^, mean (SD)	23.97 (3.48)	24.04 (3.73)	23.93 (3.63)	23.96 (3.38)	23.97 (3.51)
Lumbar spine images					
Anteroposterior	5,024	628	628	628	6,908
Lateral	5,024	628	628	628	6,908
T-score, mean L1–L4	−1.80	−1.86	−1.80	−1.92	−1.82
BMD categories, *n* (%)					
Normal	1,442 (28.7)	180 (28.6)	191 (30.4)	191 (30.4)	2,004 (29.0)
Osteopenia	1,925 (38.3)	224 (35.7)	226 (36.0)	226 (36.0)	2,601 (37.7)
Osteoporosis	1,657 (33.0)	224 (35.7)	211 (33.6)	211 (33.6)	2,302 (33.3)

Categorical and continuous data were expressed as n (%) and mean (standard deviation, SD), respectively. BMI, body mass index.

According to the DXA-based BMD screening reference standard, all patients were classified into three categories: osteoporosis (*n* = 2,302, 33.3%), osteopenia (*n* = 2,601, 37.7%), and normal (*n* = 2,004, 29.0%). In the training cohort, validation cohort, test cohort 1, and test cohort 2, 38.3%, 35.7%, 36.0%, and 36.0% of patients are osteopenic, and 33.0%, 35.7%, 33.6%, and 33.6% patients are osteoporotic, respectively.

### The consistency analysis of the delineated ROIs

One hundred cases were randomly selected and assigned to six radiologists with 4–8 years’ experience for delineating the ROI synchronously. As to the same case, the area of overlap between ROIs drawn by every two radiologists was calculated respectively. Then, the overlapping ratio was calculated. The specific calculation method of the overlap rate is that the area of overlap is divided by the combined area of the two regions. Results showed that in these 100 cases, the overlapping ratios between each two radiologists were greater than 90%.

### Performance of the CNN models with images input alone

**Figure 3:**
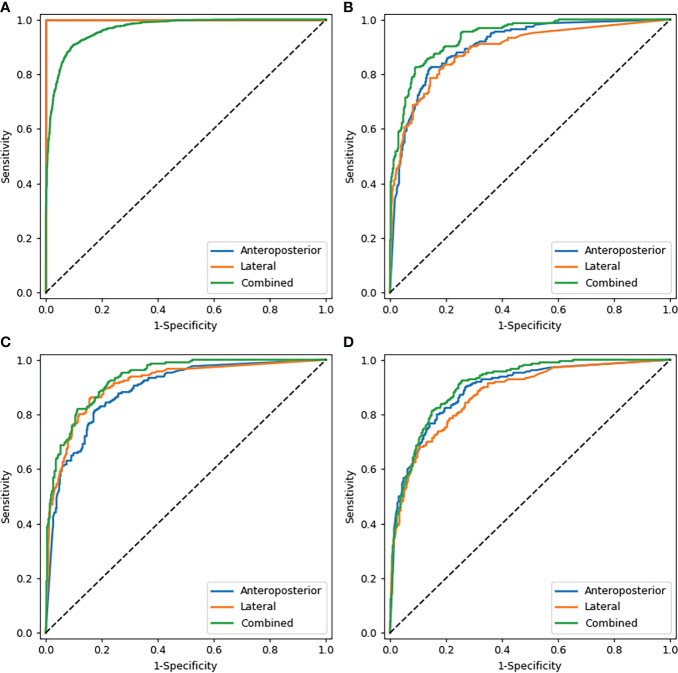
Comparison of ROC curves of the CNN models with images alone. **(A–D)** show the models that diagnosed osteoporosis in the training cohort, validation cohort, test cohort 1, and test cohort 2 respectively. Note: In the training cohort **(A)**, since AP and LAT have the same AUC values, the blue line overlaps with the orange line.

**Table 2** shows the results of the CNN model in diagnosing osteoporosis on the basis of LS x-ray images. Among the validation cohort and two test cohorts, the models based on the AP+LAT channel for diagnosing osteoporosis achieve the best performance, with an AUC range from 0.909 to 0.937, a sensitivity range from 81.90% to 84.82%, a specificity range from 82.54% to 86.63%, and a negative predictive value range from 90.08% to 91.15%. Comparison of ROC curves was performed between the CNN models constructed with single and combined image programs (**Figure 3**). The classification confusion matrices of models based on the AP+LAT channel, which report the number of true-positive, false-positive, true-negative, and false-negative results, are shown in **Table 3**.

**Table 2:** Performance of the CNN model with images inputting for classifying osteoporosis, assessed on the training, validation, and test cohorts.

Datasets	Image projection	AUC (95% CI)	Sensitivity (%)	Specificity (%)	PPV (%)	NPV (%)
Training	AP	0.996 (0.994–0.998)	99.94 (99.61–100)	99.94 (99.76–99.99)	99.88 (99.52–99.98)	99.97 (99.81–100)
	LAT	0.996 (0.994–0.998)	99.94 (99.61–100)	99.97 (99.81–100)	99.94 (99.61–100)	99.97 (99.81–100)
	AP and LAT	0.965 (0.960–0.970)	89.99 (88.42–91.37)	90.01 (88.94–91.00)	81.63 (79.76–83.36)	94.80 (93.96–95.54)
Validation	AP	0.904 (0.877–0.925)	82.14 (76.36–86.80)	85.64 (81.75–88.84)	76.03 (70.06–81.17)	89.64 (86.05–92.41)
	LAT	0.889 (0.861–0.912)	75.45 (69.18–80.83)	85.64 (81.75–88.84)	74.45 (68.17–79.88)	86.28 (82.44–89.42)
	AP and LAT	0.937 (0.914–0.954)	84.82 (79.29–89.12)	86.63 (82.83–89.72)	77.87 (72.03–82.81)	91.15 (87.73–93.71)
Test cohort 1	AP	0.889 (0.861–0.912)	81.52 (75.47–86.38)	81.77 (77.66–85.29)	69.35 (63.15–74.95)	89.74 (86.13–92.51)
	LAT	0.911 (0.885–0.932)	80.09 (73.93–85.13)	86.09 (82.31–89.19)	74.45 (68.17–79.88)	89.53 (86.01–92.27)
	AP and LAT	0.933 (0.909–0.950)	82.94 (77.03–87.62)	85.85 (82.05–88.98)	74.79 (68.63–80.11)	90.86 (87.47–93.44)
Test cohort 2	AP	0.892 (0.864–0.915)	80.48 (74.33–85.48)	81.10 (76.94–84.67)	68.15 (61.90–73.82)	89.21 (85.54–92.06)
	LAT	0.874 (0.845–0.898)	73.81 (67.22–79.51)	81.34 (77.20–84.89)	66.52 (60.02–72.47)	86.08 (82.18–89.26)
	AP and LAT	0.909 (0.883–0.930)	81.90 (75.88–86.73)	82.54 (78.48–85.98)	70.20 (63.99–75.77)	90.08 (86.53–92.80)

AP, anteroposterior; LAT, lateral; AUC, area under the curve; PPV, positive predictive value; NPV, negative predictive value.

**Table 3:** Confusion matrices of predictions and reference standards in validation and two testing datasets based on the AP+LAT channel.

		Validation (prediction)			Test cohort 1 (prediction)			Test cohort 2 (prediction)		
		Osteoporosis	Osteopenia	Normal	Osteoporosis	Osteopenia	Normal	Osteoporosis	Osteopenia	Normal
Truth	Osteoporosis	190	34	0	175	36	0	172	36	2
	Osteopenia	50	160	14	57	144	25	61	129	19
	Normal	4	71	105	2	79	110	12	76	121

The models with images input alone achieved moderate sensitivity in classifying osteopenia in the validation cohort, in which the highest AUC achieved was 0.785 (95% CI: 0.750–0.816), with a sensitivity of 71.43% and a specificity of 74.01% (**Supplementary Table 1**). In test cohort 1 and test cohort 2, the highest AUC values were 0.778 and 0.731, respectively (**Supplementary Figure 1**).

For diagnosing the normal bone mass, the diagnostic efficiency was consistently high among the validation and two test cohorts, in which the highest AUC values were 0.929, 0.926, and 0.911, respectively (**Supplementary Table 2**).

### Performance of the CNN models integrating images with clinical parameters

Before and after the addition of clinical parameters, in test cohort 1, the AUC values of AP images were statistically different only in diagnosing osteoporosis (*p <* 0.001), while those of LAT images were statistically different in diagnosing osteoporosis (*p* = 0.047) and normal BMD (*p* = 0.009). However, the AUC values of AP+LAT images have no statistical differences in three classifications (*p* > 0.05). In test cohort 2, only the AUC values predicted by LAT images for osteoporosis and osteopenia were statistically different (*p* = 0.017 and *p <* 0.001, respectively), and the other AUC values have no statistical difference (*p* > 0.05).

**Figure 4:**
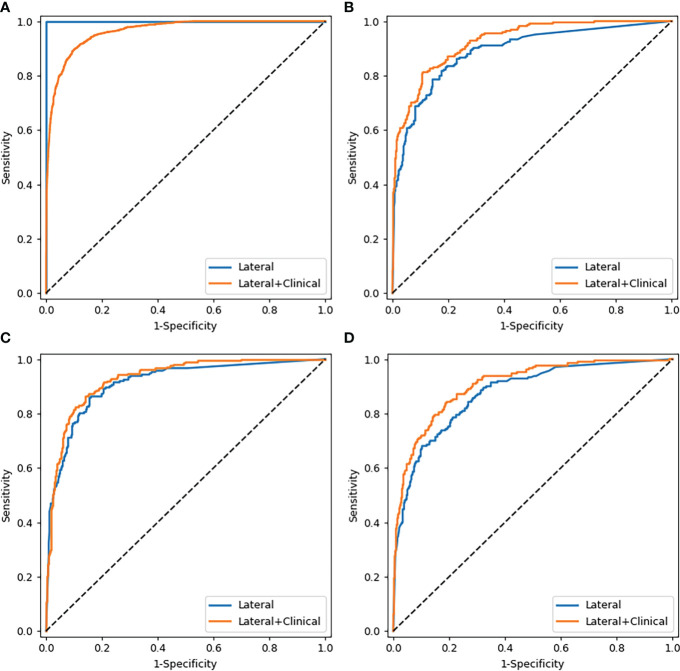
Comparison of ROC curves of the CNN models based on lateral images with and without combining clinical parameters. **(A–D)** were the curves of the training cohort, validation cohort, test cohort 1, and test cohort 2, respectively.

The performance of the proposed models that integrate images with clinical parameters has shown a slight improvement over models with AP or LAT images alone for diagnosing osteoporosis, in which the AUC increased about 2%–4%. Meanwhile, the specificity and positive predictive values improved as well (**Table 4**). In the model diagnosing osteoporosis based on the LAT channel, the AUC value and sensitivity increased in the validation cohort and test cohort 1, while in test cohort 2, the AUC and specificity have improved, but sensitivity slightly declined (from 73.81% to 70.00%). **Figure 4** demonstrates the comparison of the efficacy of the CNN models based on LAT image with and without integrating clinical parameters in diagnosing osteoporosis, accessed on the test cohort, validation cohort, and two test cohorts.

**Table 4:** Performance of the CNN model integrating images with clinical parameters inputting for classifying osteoporosis, assessed on the training, validation, and test cohorts.

Datasets	Image projection	AUC (95% CI)	Sensitivity (%)	Specificity (%)	PPV (%)	NPV (%)
Training	AP	0.981 (0.976–0.984)	86.20 (84.42–87.80)	95.75 (95.00–96.40)	90.91 (89.35–92.26)	93.36 (92.47–94.16)
	LAT	0.963 (0.957–0.968)	88.67 (87.02–90.13)	90.37 (89.31–91.34)	81.95 (80.07–83.69)	94.18 (93.30–94.95)
	AP and LAT	0.996 (0.993–0.998)	99.94 (99.61–100)	99.97 (99.81–100)	99.94 (99.61–100)	99.97 (99.81–100)
Validation	AP	0.922 (0.897–0.941)	73.21 (66.83–78.79)	92.57 (89.46–94.85)	84.54 (78.50–89.17)	86.18 (82.48–89.21)
	LAT	0.926 (0.901–0.944)	81.70 (75.87–86.41)	86.88 (83.10–89.94)	77.54 (71.58–82.59)	89.54 (85.98–92.31)
	AP and LAT	0.928 (0.904–0.947)	75.00 (68.70–80.42)	92.08 (88.89–94.44)	84.00 (78.01–88.65)	86.92 (83.26–89.89)
Test cohort 1	AP	0.928 (0.904–0.946)	73.93 (67.37–79.61)	90.17 (86.80–92.77)	79.19 (72.71–84.50)	87.24 (83.63–90.17)
	LAT	0.930 (0.907–0.949)	81.52 (75.47–86.38)	88.73 (85.20–91.52)	78.54 (72.39–83.66)	90.46 (87.09–93.05)
	AP and LAT	0.943 (0.921–0.960)	75.36 (68.87–80.90)	91.61 (88.42–94.01)	81.96 (75.66–86.96)	88.02 (84.50–90.85)
Test cohort 2	AP	0.912 (0.887–0.933)	68.57 (61.76–74.69)	92.34 (89.26–94.63)	81.82 (75.15–87.06)	85.40 (81.72–88.45)
	LAT	0.905 (0.878–0.926)	70.00 (63.24–76.01)	88.76 (85.24–91.54)	75.77 (69.01–81.50)	85.48 (81.73–88.59)
	AP and LAT	0.915 (0.889–0.935)	69.05 (62.25–75.13)	92.58 (89.53–94.83)	82.39 (75.77–87.55)	85.62 (81.96–88.65)

AP, anteroposterior; LAT, lateral; AUC, area under the curve; PPV, positive predictive value; NPV, negative predictive value.

Regarding categorizing osteopenia and normal bone mass, the proposed deep learning model integrating images with clinical parameters also outperformed the models with images inputting alone in test cohorts (**Supplementary Tables 3**, **4**), in which the sensitivity increased particularly.

## Discussion

In this multicenter study, we developed a deep learning method based on conventional lumbar spine DR examinations performed for other clinical symptoms, intended to diagnose osteoporosis and osteopenia in postmenopausal women and men over 50 years. Our results revealed that the deep learning method has the prospect of automatic BMD categorization in clinical practice. Moreover, another finding was obtained: the model combining lumbar images with clinical information could improve the performance, particularly based on the LAT channel.

Deep learning uses neural networks as framework, and is performed *via* multiple abstraction layers (30–32). CNN is one of the most common deep learning algorithms; the processing of information is performed by the brain’s neurons, which is specialized in handling a large amount of inputs. In this study, we employed DenseNet that connected each layer to every other layer in a feed-forward pattern, requiring less computation to achieve high performance (29). Based on it, we trained a CNN model to evaluate the bone mass in postmenopausal women and men over 50 years old. Our classification models were built on the triple classification of the L1–L4 LS x-ray images divided into normal, osteopenia, and osteoporosis, which differed from general deep learning models on the basis of binary classification. As a screening method for a disease, the high sensitivity of models reduces false-negative categories; therefore, the osteoporotic individuals will be recognized probably and treated accordingly. In our research, sensitivity of the models diagnosing osteoporosis was high among validation and two testing datasets (≥81.90% based on the AP+LAT channel). However, the AUC and sensitivity of the models classifying osteopenia were slightly low, which may be attributed to data imbalance, and the ROI of the LS images (including partial vertebral osteophyte and spinous process in LAT image) input to models distinct from DXA (excluding vertebral osteophyte). The inputting images of models including partial vertebral osteophyte will lead to overestimating bone mass, but somewhat reducing the sensitivity of osteopenia and normal. Moreover, the models have a triple classification, and the T-score of osteopenia was between osteoporosis and normal; thus, part of osteopenia may be classified as osteoporosis and normal. Thus, the diagnostic efficiency of the model for classifying osteopenia was lower than the normal. Above all, we analyzed that a considerable percentage of patients enrolled in our research was at the critical point of osteopenia and normal bone mass. Thus, further study is aimed to improve the models’ sensitivity in the diagnosis of osteopenia.

Osteoporosis is a major health disease with the increase in the aging population, affecting post-menopausal women most frequently, and is gradually considered as a clinical problem among elderly men. In their life, about 50% of women and 20% of men will suffer from osteoporotic fracture (33). The risk of subsequent fractures following an initial fracture is increased and the adjusted hazard ratios were higher in men than in women (34). However, few studies have analyzed osteoporosis in men. On account of this, men aged ≥50 years were also included in our study. Furthermore, majority of the studies demonstrated that advancing age, gender, and low body weight were the additional risk factors of fracture for both men and women (35, 36). Thus, we obtained the models combined LS radiographs with age, gender, and BMI, to evaluate whether the clinical variables would affect the effectiveness of the CNN models in categorizing osteoporosis and osteopenia. The results revealed that it was helpful to improve the sensitivity of models classifying osteopenia, but it did not have much significance in classifying osteoporosis or the BMD. The main reason may be that the sensitivity of models in categorizing osteoporosis was comparatively high, and the deep learning is mainly about automatically acquiring the internal features of images.

Summarily, our study has several strengths. First, this research is a multicenter study with a large data volume, including internal and external validation; hence, the results are relatively stable. Zhang et al. (13) constructed a deep CNN model to classify osteoporosis and osteopenia that is based on the AP and LAT LS radiographs of 808 postmenopausal women. Their model diagnosing osteoporosis achieved an AUC of 0.767 with a sensitivity of 73.7%. In contrast to the previous study (13), the AUC (0.93 vs. 0.77) and sensitivity (82.9% vs. 73.7%) of our models in the diagnosis of osteoporosis improved significantly. Second, the groups of study included not only postmenopausal women but also men over 50 years old. The AI model is more applicable to clinical practice due to the completeness of subjects. Third, our model could diagnose osteoporosis and osteopenia through image features extracted from conventional lumbar radiographs. Thus, this method has the potential to be applied in detecting osteoporosis and osteopenia for many “opportunistic screening” without additional costs.

There are also some potential limitations to this study. Firstly, the retrospective inclusion of subjects who underwent paired LS radiographs and DXA examinations may have led to selection bias. Secondly, DXA examinations could not eliminate the effect of cortex, hyperosteogeny, and arteriosclerosis sclerosis on BMD measurement (11), which might underestimate the actual loss of bone mass. Similarly, the proposed method may also be influenced by aortic sclerosis, bowel gas, and osteophytic spurs, which may cause overestimating BMD values. Moreover, individuals who suffered from lumbar vertebra tumor, inflammatory diseases, serious scoliosis, or deformity were not appropriate for the CNN models as well. Thirdly, all the ROIs were delineated manually, which was time-consuming though it was relatively accurate. Fourthly, women or men under 50 years old were not included in this study. Therefore, the application of our results to these populations is limited. Lastly, the developed deep learning models could not predict the exact fracture risk of individuals, and it needs further study.

## Conclusions

In conclusion, our research showed that the proposed deep learning models based on routine lumbar spine radiographs obtained for other reasons attained favorable performance on BMD classification in men and postmenopausal women aged ≥50, which would be an available tool for clinicians in opportunistic osteoporosis screening without additional radiation exposure or cost. It could be applied in the circumstance that lumbar spine radiograph is available but DXA examination is lacking, and it is especially suitable for patients with physical examination. Early detection of osteoporosis and osteopenia is beneficial to identify those at risk of fracture and provide treatment to prevent further losses.

## Data availability statement

The original contributions presented in the study are included in the article/**Supplementary Material**. Further inquiries can be directed to the corresponding author.

## Ethics statement

This study was reviewed and approved by the institutional review board and the ethics committee of the host hospital (Ethics Committee of Guangdong Provincial Hospital of Chinese Medicine ZE2020-299-01). The requirement for written informed consent was waived due to the retrospective nature of the study.

## Author contributions

All authors contributed to the article and approved the submitted version. Study concept and design: BL and LM. Acquisition of data: LM, ZX, and XL. Analysis of data: ZL, GL, HW and ZY. Drafting of the manuscript: LM, LP and BL. Critical revision: JC and XL. Statistical analysis: LP and LM.

## Funding

This work was supported by the following projects: 1. Traditional Chinese Medicine Science and Technology Project of Guangdong Hospital of Traditional Chinese Medicine (YN2020MS09). 2. Science and Technology Program of Guangzhou (202102010260).

## Conflict of interest

LP was employed by Guangzhou YLZ Ruitu Information Technology Co, Ltd.

The remaining authors declare that the research was conducted in the absence of any commercial or financial relationships that could be construed as a potential conflict of interest.

## Publisher’s note

All claims expressed in this article are solely those of the authors and do not necessarily represent those of their affiliated organizations, or those of the publisher, the editors and the reviewers. Any product that may be evaluated in this article, or claim that may be made by its manufacturer, is not guaranteed or endorsed by the publisher.

## References

[1] Lee JJ Aghdassi E Cheung AM Morrison S Cymet A Peeva V . Ten-year absolute fracture risk and hip bone strength in Canadian women with systemic lupus erythematosus. J Rheumatol (2012) 39(7):1378–84. doi: 10.3899/jrheum.111589 22660811

[2] Cui Z Meng X Feng H Zhuang S Liu Z Zhu T . Estimation and projection about the standardized prevalence of osteoporosis in mainland China. Arch Osteoporos (2019) 15(1):2. doi: 10.1007/s11657-019-0670-6 31811461

[3] Zeng Q Li N Wang Q Feng J Sun D Zhang Q . The prevalence of osteoporosis in China, a nationwide, multicenter DXA survey. J Bone Miner Res (2019) 34(10):1789–97. doi: 10.1002/jbmr.3757 31067339

[4] Schuit SCE van der Klift M Weel AEAM de Laet CEDH Burger H Seeman E . Fracture incidence and association with bone mineral density in elderly men and women: the Rotterdam study. Bone (2004) 34(1):195–202. doi: 10.1016/j.bone.2003.10.001 14751578

[5] Wainwright SA Marshall LM Ensrud KE Cauley JA Black DM Hillier TA . Hip fracture in women without osteoporosis. J Clin Endocrinol Metab (2005) 90(5):2787–93. doi: 10.1210/jc.2004-1568 15728213

[6] Brown C . Osteoporosis: Staying strong. Nature (2017) 550(7674):S15–7. doi: 10.1038/550S15a 28976955

[7] Liow MHL Ganesan G Chen JDY Koh JSB Howe TS Yong EL . Excess mortality after hip fracture: fracture or pre-fall comorbidity? Osteoporosis Int (2021) 32(12):2485–92. doi: 10.1007/s00198-021-06023-0 34129060

[8] Dimai HP . Use of dual-energy X-ray absorptiometry (DXA) for diagnosis and fracture risk assessment; WHO-criteria, T- and z-score, and reference databases. Bone (2017) 104:39–43. doi: 10.1016/j.bone.2016.12.016 28041872

[9] Choi YJ . Dual-energy X-ray absorptiometry: Beyond bone mineral density determination. Endocrinol Metab (Seoul Korea) (2016) 31(1):25–30. doi: 10.3803/EnM.2016.31.1.25 PMC4803557 26996419

[10] China NHCO . Epidemiological investigation of osteoporosis in China (2021). Available at: http://www.phsciencedata.cn/Share/jsp/PublishManager/foregroundView/1/9eaadbb3-bd64-4531-9d9f-753ec183f26d.

[11] Ito M Hayashi K Yamada M Uetani M Nakamura T . Relationship of osteophytes to bone mineral density and spinal fracture in men. Radiology (1993) 189(2):497–502. doi: 10.1148/radiology.189.2.8210380 8210380

[12] Chang G Honig S Brown R Deniz CM Egol KA Babb JS . Finite element analysis applied to 3-T MR imaging of proximal femur microarchitecture: lower bone strength in patients with fragility fractures compared with control subjects. Radiology (2014) 272(2):464–74. doi: 10.1148/radiol.14131926 PMC4263634 24689884

[13] Zhang B Yu K Ning Z Wang K Dong Y Liu X . Deep learning of lumbar spine X-ray for osteopenia and osteoporosis screening: A multicenter retrospective cohort study. Bone (2020) 140:115561. doi: 10.1016/j.bone.2020.115561 32730939

[14] Chartrand G Cheng PM Vorontsov E Drozdzal M Turcotte S Pal CJ . Deep learning: A primer for radiologists. Radiographics (2017) 37(7):2113–31. doi: 10.1148/rg.2017170077 29131760

[15] Yasaka K Akai H Kunimatsu A Kiryu S Abe O . Deep learning with convolutional neural network in radiology. Jpn J Radiol (2018) 36(4):257–72. doi: 10.1007/s11604-018-0726-3 29498017

[16] Yasaka K Akai H Abe O Kiryu S . Deep learning with convolutional neural network for differentiation of liver masses at dynamic contrast-enhanced CT: A preliminary study. Radiology (2018) 286(3):887–96. doi: 10.1148/radiol.2017170706 29059036

[17] Kiryu S Yasaka K Akai H Nakata Y Sugomori Y Hara S . Deep learning to differentiate parkinsonian disorders separately using single midsagittal MR imaging: a proof of concept study. Eur Radiol (2019) 29(12):6891–9. doi: 10.1007/s00330-019-06327-0 31264017

[18] Larson DB Chen MC Lungren MP Halabi SS Stence NV Langlotz CP . Performance of a deep-learning neural network model in assessing skeletal maturity on pediatric hand radiographs. Radiology (2018) 287(1):313–22. doi: 10.1148/radiol.2017170236 29095675

[19] Urakawa T Tanaka Y Goto S Matsuzawa H Watanabe K Endo N . Detecting intertrochanteric hip fractures with orthopedist-level accuracy using a deep convolutional neural network. Skeletal Radiol (2019) 48(2):239–44. doi: 10.1007/s00256-018-3016-3 29955910

[20] Derkatch S Kirby C Kimelman D Jozani MJ Davidson JM Leslie WD . Identification of vertebral fractures by convolutional neural networks to predict nonvertebral and hip fractures: A registry-based cohort study of dual X-ray absorptiometry. Radiology (2019) 293(2):405–11. doi: 10.1148/radiol.2019190201 31526255

[21] Cheng CT Ho TY Lee TY Chang CC Chou CC Chen CC . Application of a deep learning algorithm for detection and visualization of hip fractures on plain pelvic radiographs. Eur Radiol (2019) 29(10):5469–77. doi: 10.1007/s00330-019-06167-y PMC6717182 30937588

[22] dLee KS Jung SK Ryu JJ Shin SW Choi J . Evaluation of transfer learning with deep convolutional neural networks for screening osteoporosis in dental panoramic radiographs. J Clin Med (2020) 9(2):392–405. doi: 10.3390/jcm9020392 PMC7074309 32024114

[23] Lee S Choe EK Kang HY Yoon JW Kim HS . The exploration of feature extraction and machine learning for predicting bone density from simple spine X-ray images in a Korean population. Skeletal Radiol (2020) 49(4):613–8. doi: 10.1007/s00256-019-03342-6 31760458

[24] Tecle N Teitel J Morris MR Sani N Mitten D Hammert WC . Convolutional neural network for second metacarpal radiographic osteoporosis screening. J Handb Surg Am (2020) 45(3):175–81. doi: 10.1016/j.jhsa.2019.11.019 31959378

[25] Hussain D Han SM . Computer-aided osteoporosis detection from DXA imaging. Comput Methods Programs BioMed (2019) 173:87–107. doi: 10.1016/j.cmpb.2019.03.011 31046999

[26] Valentinitsch A Trebeschi S Kaesmacher J Lorenz C Loffler MT Zimmer C . Opportunistic osteoporosis screening in multi-detector CT images *via* local classification of textures. Osteoporos Int (2019) 30(6):1275–85. doi: 10.1007/s00198-019-04910-1 PMC6546649 30830261

[27] Fang Y Li W Chen X Chen K Kang H Yu P . Opportunistic osteoporosis screening in multi-detector CT images using deep convolutional neural networks. Eur Radiol (2021) 31(4):1831–42. doi: 10.1007/s00330-020-07312-8 33001308

[28] Camacho PM Petak SM Binkley N Diab DL Eldeiry LS Farooki A . American Association of clinical endocrinologists/American colloege of endocrinology clinical practice guidelines for the diagnosis and treatment and of postmenopausal osteoporosis-2020 update executive summary. Endocr Pract (2020) 26(5):564–70. doi: 10.4158/GL-2020-0524 32427525

[29] Huang G Liu Z Van Der Maaten L Weinberger KQ . Densely connected convolutional networks, IEEE Computer Society (2017) 24(5):2261–9. doi: 10.1109/CVPR.2017.243

[30] Le QV . A tutorial on deep learning part 1: Nonlinear classifiers and the back propagation algorithm. Mountain View, CA: Google Inc (2015).

[31] Le QV . A tutorial on deep learning part 2: autoencoders, convolutional neural networks and recurrent neural networks. Mountain View, CA: Google Inc (2015).

[32] Lecun Y Bengio Y Hinton G . Deep learning. Nature (2015) 521(7553):436–44. doi: 10.1038/nature14539 26017442

[33] Force USPS . Screening for osteoporosis in postmenopausal women: Recommendations and rationale. Am Fam Med (2002) 137(6):526–8. doi: 10.7326/0003-4819-137-6-200209170-00014 12230355

[34] Morin SN Yan L Lix LM Leslie WD . Long-term risk of subsequent major osteoporotic fracture and hip fracture in men and women: a population-based observational study with a 25-year follow-up. Osteoporos Int (2021) 32(12):2525–32. doi: 10.1007/s00198-021-06028-9 34165587

[35] Anpalahan M Morrison SG Gibson SJ . Hip fracture risk factors and the discriminability of hip fracture risk vary by age: a case-control study. Geriatr Gerontol Int (2014) 14(2):413–9. doi: 10.1111/ggi.12117 23879545

[36] Curry SJ Krist AH Owens DK Barry MJ Caughey AB Davidson KW . Screening for osteoporosis to prevent fractures US preventive services task force recommendation statement. JAMA-J Am Med Assoc (2018) 319(24):2521–31. doi: 10.1001/jama.2018.7498 29946735

