# Supplementary material for: Deep learning for screening primary osteopenia and osteoporosis using spine radiographs and patient clinical covariates in a Chinese population
Source: Front Endocrinol (Lausanne). 2022 Sep 13;13:971877. doi: 10.3389/fendo.2022.971877 (PMC9513384; doi:10.3389/fendo.2022.971877)
Supplement: Supplementary file 1 [file DataSheet_1.zip › supplementary material.docx]

**Supplementary materials**

**Supplementary Table 1**

Performance of the CNN model with images inputting for classifying osteopenia, assessed on the training, validation and test cohorts.

| Datasets | Image  projection | AUC  (95%CI) | Sensitivity  (%) | Specificity  (%) | PPV  (%) | NPV  (%) |
| --- | --- | --- | --- | --- | --- | --- |
| Training cohort | AP | 1.000  (0.999-1.000) | 99.90  (99.58-99.98) | 99.97  (99.79-100) | 99.95  (99.66-100) | 99.94  (99.74-99.99) |
|  | LAT | 0.996  (0.994-0.998) | 99.95  (99.66-100) | 99.97  (99.79-100) | 99.95  (99.66-100) | 99.97  (99.79-100) |
|  | AP and LAT | 0.875  (0.866-0.884) | 79.39  (77.49-81.16) | 80.86  (79.42-82.22) | 71.97  (69.99-73.86) | 86.37  (85.05-87.58) |
| Validation cohort | AP | 0.765  (0.729-0.797) | 59.82  (53.06-66.24) | 82.67  (78.55-86.16) | 65.69  (58.68-72.09) | 78.77  (74.51-82.51) |
|  | LAT | 0.761 (0.726-0.79)4 | 61.61  (54.87-67.94) | 75.50  (70.94-79.55) | 58.23  (51.65-64.53) | 78.01  (73.50-81.95) |
|  | AP and LAT | 0.785  (0.750-0.816) | 71.43  (64.96-77.15) | 74.01  (69.39-78.16) | 60.38  (54.19-66.26) | 82.37  (77.97-86.07) |
| Test cohort1 | AP | 0.743 (0.707-0.777) | 51.77  (45.06-58.42) | 82.34  (78.18-85.87) | 62.23  (54.85-69.10) | 75.23  (70.87-79.14) |
|  | LAT | 0.778  (0.743-0.809) | 58.41  (51.67-64.85) | 80.10  (75.79-83.82) | 62.26  (55.34-68.74) | 77.40  (73.02-81.27) |
|  | AP and LAT | 0.757  (0.721-0.789) | 63.72  (57.04-69.92) | 71.39  (66.66-75.71) | 55.60  (49.32-61.71) | 77.78  (73.12-81.85) |
| Test cohort2 | AP | 0.731  (0.695-0.765) | 45.93  (39.08-52.94) | 79.00  (74.72-82.74) | 52.17  (44.72-59.54) | 74.55  (70.18-78.48) |
|  | LAT | 0.718 (0.681-0.752) | 52.63  (45.64-59.53) | 73.51  (68.96-77.62) | 49.77  (43.02-56.54) | 75.68  (71.15-79.71) |
|  | AP and LAT | 0.722 (0.684-0.756) | 61.72  (54.74-68.27) | 73.27  (68.71-77.39) | 53.53  (47.02-59.92) | 79.33  (74.88-83.18) |

Note: AP: anteroposterior, LAT: lateral, AUC: area under the curve, PPV: positive predictive value, NPV: negative predictive value.

**Supplementary Figure 1**


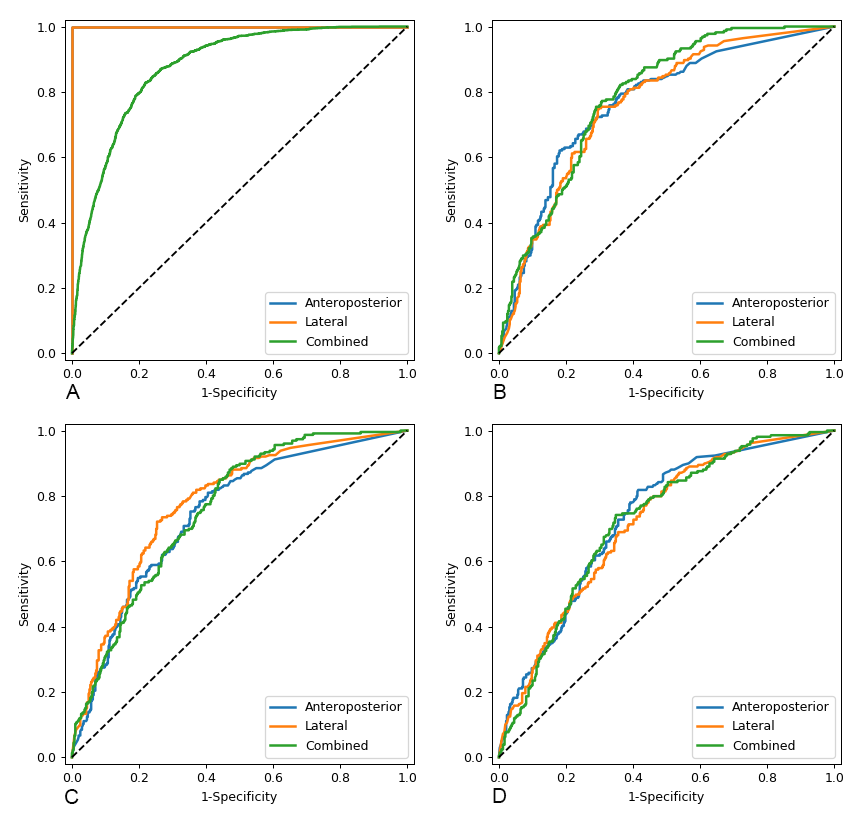


Comparison of ROC curves of the CNN models based on single and combined image programs. **(A-D)** shows the curves of models diagnosing the osteopenia in the training cohort, validation cohort, test cohort 1 and test cohort 2, respectively.

**Supplementary Table 2**

Performance of the CNN model with images inputting for classifying the normal BMD, assessed on the training, validation and test cohorts.

| Datasets | Image  projection | AUC  (95%CI) | Sensitivity  (%) | Specificity  (%) | PPV  (%) | NPV  (%) |
| --- | --- | --- | --- | --- | --- | --- |
| Training cohort | AP | 0.996 (0.994-0.998) | 99.93  (99.55-100) | 99.97  (99.82-100) | 99.93  (99.55-100) | 99.97  (99.82-100) |
|  | LAT | 0.996  (0.994-0.998) | 99.93  (99.55-100) | 99.97  (99.82-100) | 99.93  (99.55-100) | 99.97  (99.82-100) |
|  | AP and LAT | 0.966  (0.960-0.971) | 68.91  (66.44-71.27) | 97.74  (97.18-98.19) | 92.47  (90.69-93.94) | 88.63  (87.59-89.59) |
| Validation cohort | AP | 0.909  (0.883-0.930) | 77.78  (70.86-83.48) | 90.62  (87.45-93.09) | 76.92  (69.99-82.69) | 91.03  (87.89-93.44) |
|  | LAT | 0.929  (0.906-0.948) | 72.22  (64.98-78.50) | 92.41  (89.46-94.61) | 79.27  (72.10-85.03) | 89.22  (85.95-91.82) |
|  | AP and LAT | 0.928  (0.904-0.946) | 58.33  (50.75-65.55) | 96.88  (94.69-98.21) | 88.24  (80.73-93.18) | 85.27  (81.82-88.17) |
| Test cohort1 | AP | 0.910  (0.884-0.930) | 75.92  (69.10-81.67) | 89.24  (85.86-91.92) | 75.52  (68.70-81.30) | 89.45  (86.09-92.10) |
|  | LAT | 0.907  (0.881-0.928) | 74.35  (67.44-80.25) | 89.24  (85.86-91.92) | 75.13  (68.23-80.99) | 88.84  (85.42-91.55) |
|  | AP and LAT | 0.926  (0.902-0.945) | 57.59  (50.24-64.63) | 94.28  (91.56-96.19) | 81.48  (73.68-87.44) | 83.57  (79.93-86.67) |
| Test cohort2 | AP | 0.911  (0.885-0.931) | 71.77  (65.07-77.66) | 89.02  (85.53-91.77) | 76.53  (69.85-82.15) | 86.34  (82.66-89.37) |
|  | LAT | 0.897  (0.870-0.919) | 64.11  (57.17-70.53) | 90.45  (87.13-93.01) | 77.01  (69.91-82.89) | 83.48  (79.67-86.71) |
|  | AP and LAT | 0.898  (0.871-0.920) | 57.89  (50.88-64.62) | 94.99  (92.32-96.79) | 85.21  (78.05-90.41) | 81.89  (78.11-85.16) |

Note: BMD: bone mass density, AP: anteroposterior, LAT: lateral, AUC: area under the curve, PPV: positive predictive value, NPV: negative predictive value.

**Supplementary Table 3**

Performance of the developed CNN model integrating images with clinical parameters for classifying osteopenia, assessed on the training, validation and test cohorts

| Datasets | Image  projection | AUC  (95%CI) | Sensitivity  (%) | Specificity  (%) | PPV  (%) | PPV  (%) |
| --- | --- | --- | --- | --- | --- | --- |
| Training cohort | AP | 0.941  (0.934-0.948) | 84.44  (82.72-86.01) | 87.33  (86.10-88.47) | 80.50  (78.68-82.19) | 90.06  (88.93-91.10) |
|  | LAT | 0.883  (0.874-0.892) | 75.22  (73.21-77.13) | 84.05  (82.70-85.31) | 74.48  (72.47-76.40) | 84.57  (83.23-85.81) |
|  | AP and LAT | 0.996  (0.994-0.998) | 99.95  (99.66-100) | 99.97  (99.79-100) | 99.95  (99.66-100) | 99.97  (99.79-100) |
| Validation cohort | AP | 0.792  (0.758-0.823) | 73.66  (67.29-79.20) | 72.77  (68.10-77.00) | 60.00  (53.93-65.79) | 83.29  (78.89-86.94) |
|  | LAT | 0.792  (0.758-0.823) | 69.64  (63.10-75.50) | 76.73  (72.24-80.70) | 62.40  (56.05-68.36) | 82.01  (77.68-85.67) |
|  | AP and LAT | 0.781  (0.746-0.812) | 64.73  (58.05-70.90) | 78.47  (74.07-82.31) | 62.50  (55.90-68.68) | 80.05  (75.70-83.81) |
| Test cohort 1 | AP | 0.778  (0.743-0.810) | 65.04  (58.40-71.17) | 73.88  (69.25-78.05) | 58.33  (51.97-64.44) | 78.99  (74.45-82.93) |
|  | LAT | 0.791  (0.757-0.822) | 66.81  (60.21-72.83) | 78.86  (74.47-82.68) | 63.98  (57.46-70.04) | 80.87  (76.55-84.57) |
|  | AP and LAT | 0.783  (0.748-0.814) | 63.27  (56.59-69.50) | 78.36  (73.94-82.22) | 62.17  (55.53-68.40) | 79.15  (74.75-82.96) |
| Test cohort 2 | AP | 0.760  (0.724-0.792) | 66.51  (59.62-72.78) | 72.55  (67.97-76.72) | 54.72  (48.38-60.92) | 81.28  (76.88-85.03) |
|  | LAT | 0.796  (0.762-0.826) | 70.33  (63.57-76.34) | 73.27  (68.71-77.39) | 56.76  (50.47-62.84) | 83.20  (78.90-86.79) |
|  | AP and LAT | 0.751  (0.715-0.784) | 64.11  (57.17-70.53) | 75.42  (70.95-79.41) | 56.54  (49.96-62.90) | 80.82  (76.49-84.53) |

Note: AP: anteroposterior, LAT: lateral, AUC: area under the curve, PPV: positive predictive value, NPV: negative predictive value.

**Supplementary Table 4**

Performance of the developed CNN model integrating images with clinical parameters for classifying the normal BMD, assessed on the training, validation and test cohorts.

| Datasets | Image  projection | AUC  (95%CI) | Sensitivity  (%) | Specificity  (%) | PPV  (%) | PPV  (%) |
| --- | --- | --- | --- | --- | --- | --- |
| Training cohort | AP | 0.980  (0.975-0.983) | 87.95  (86.13-89.56) | 95.36  (94.61-96.02) | 88.44  (86.65-90.02) | 95.15  (94.38-95.82) |
|  | LAT | 0.958  (0.952-0.963) | 77.15  (74.88-79.27) | 95.11  (94.34-95.78) | 86.42  (84.40-88.22) | 91.16  (90.20-92.05) |
|  | AP and LAT | 0.996  (0.994-0.998) | 99.93  (99.55-100) | 99.97  (99.82-100) | 99.93  (99.55-100) | 99.97  (99.82-100) |
| Validation cohort | AP | 0.918  (0.893-0.938) | 70.56  (63.23-76.98) | 92.86  (89.96-94.99) | 79.87  (72.63-85.64) | 88.70  (85.40-91.35) |
|  | LAT | 0.937  (0.915-0.954) | 67.22  (59.78-73.91) | 95.31  (92.81-97.00) | 85.21  (78.05-90.41) | 87.86  (84.54-90.56) |
|  | AP and LAT | 0.948  (0.927-0.964) | 80.56  (73.86-85.92) | 88.62  (85.22-91.33) | 73.98  (67.15-79.85) | 91.90 (88.81-94.22) |
| Test cohort 1 | AP | 0.920  (0.895-0.939) | 71.20  (64.14-77.40) | 90.16  (86.88-92.71) | 75.98  (68.92-81.90) | 87.75  (84.27-90.57) |
|  | LAT | 0.935  (0.911-0.952) | 73.82  (66.89-79.78) | 92.68  (89.71-94.86) | 81.50  (74.74-86.83) | 89.01  (85.68-91.66) |
|  | AP and LAT | 0.939  (0.917-0.956) | 80.10  (73.59-85.38) | 88.33  (84.85-91.11) | 75.00  (68.37-80.66) | 91.04  (87.81-93.50) |
| Test cohort 2 | AP | 0.899  (0.872-0.921) | 69.38  (62.58-75.45) | 87.35  (83.69-90.30) | 73.23  (66.40-79.14) | 85.12  (81.32-88.27) |
|  | LAT | 0.908  (0.880-0.929) | 68.90  (62.08-75.01) | 92.60  (89.55-94.84) | 82.29  (75.64-87.48) | 85.65  (82.00-88.68) |
|  | AP and LAT | 0.915  (0.889-0.935) | 76.56  (70.11-82.00) | 86.87  (83.17-89.88) | 74.42  (67.95-80.00) | 88.14  (84.53-91.01) |

Note: BMD: bone mass density, AP: anteroposterior, LAT: lateral, AUC: area under the curve, PPV: positive predictive value, NPV: negative predictive value.
