# Supplementary figures and images for: Deep learning for screening primary osteopenia and osteoporosis using spine radiographs and patient clinical covariates in a Chinese population
Source: Front Endocrinol (Lausanne). 2022 Sep 13;13:971877. doi: 10.3389/fendo.2022.971877 (PMC9513384; doi:10.3389/fendo.2022.971877)

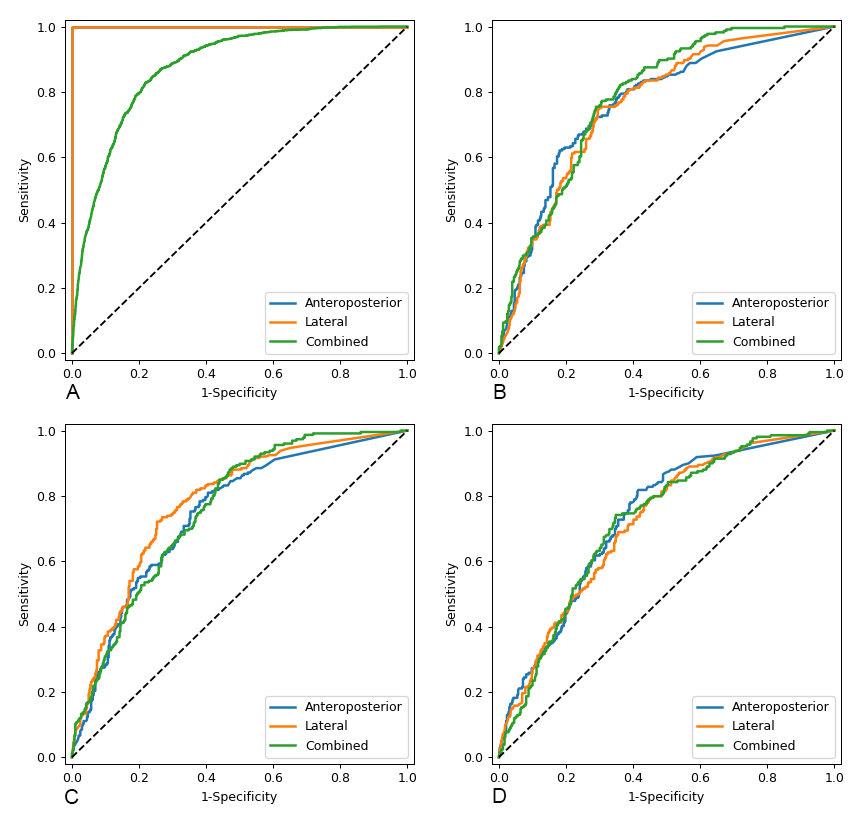

Supplement: Supplementary file 1 [file DataSheet_1.zip › Supplement Figure1.tif]
